# CAREGIVER PHYSICAL HEALTH AND RISK OF ABUSE AND NEGLECT AMONG PERSONS LIVING WITH DEMENTIA

**DOI:** 10.1093/geroni/igae098.2351

**Published:** 2024-12-31

**Authors:** Henrietta Bennett, Wesley Browning, Mustafa Yildiz, Carolyn Pickering

**Affiliations:** University of Alabama at Birmingham, Birmingham, Alabama, United States; The University of Texas Health Science Center Houston, Houston, Texas, United States; The University of Texas Health Houston, Houston, Texas, United States; The University of Texas Health Houston, Houston, Texas, United States

## Abstract

Dementia family caregivers face unique challenges that increase their risk for poor physical health. Caregivers with worse physical health may have difficulty completing caregiving tasks, placing their care recipients at risk of negative health outcomes. The purpose of this study was to investigate the association of caregivers’ satisfaction with daily sleep quality and self-reported global health and the daily risk of engaging in abusive and neglectful behaviors towards their care recipient. Using a micro-longitudinal approach, the study included 453 family caregivers who lived with and provided unpaid care to a family member living with dementia. Results from Generalized Linear Mixed models revealed that caregivers who reported being more satisfied with their sleep quality than normal on a given day had a 23% lower likelihood of engaging in physically aggressive behaviors (OR = 0.77, p <.05) and a 20% lower likelihood of engaging in psychologically aggressive behaviors that same day (OR = 0.80, p <.001). Similarly, better than average sleep satisfaction on a given day was found to have a protective effect against neglect, with a 9% reduction in the likelihood of engaging in neglectful behaviors (OR = 0.91, p <.01). Caregiver self-reported health did not affect daily odds of abusive and neglectful behaviors. These findings identify sleep satisfaction as a modifiable daily protective factor against elder abuse and neglect and suggest a need for adaptation and implementation of sleep interventions for dementia family caregivers.

